# Supplementary material for: Dynalign II: common secondary structure prediction for RNA homologs with domain insertions
Source: Nucleic Acids Res. 2014 Nov 21;42(22):13939–48. doi: 10.1093/nar/gku1172 (PMC4267632; doi:10.1093/nar/gku1172)
Supplement: SUPPLEMENTARY DATA [file supp_gku1172_nar-02021-z-2014-File012.zip › manual/Text/Fold.html]

RNAstructure Command Line Help: Fold


|  |  |  |
| --- | --- | --- |
|  | RNAstructure Command Line Help  Fold and Fold-smp | - Contents - Index |
| Fold is used to predict the lowest free energy structure and a set of suboptimal structures, i.e. low free energy structures, using a variety of constraints. Fold-smp is a parallel processing version for use on multi-core computers, built using OpenMP. USAGE: Fold <seq file> <ct file> [options] OR: Fold-smp <seq file> <ct file> [options] Required parameters: <seq file> \| The name of a sequence file containing input data. Note that lowercase nucleotides are forced single-stranded in structure prediction.; <ct file> \| The name of a CT file to which output will be written. Options which don't require added values: -d, -D, --DNA \| Specify that the sequence is DNA, and DNA parameters are to be used. Default is to use RNA parameters.; -h, -H, --help \| Display the usage details message. Options which require added values: -c, -C, --constraint \| Specify a folding constraints file to be applied. For Fold, current supported constraints are force pairs, force a nucleotide to be single stranded, and force a nucleotide to be double stranded. Default is to have no constraints applied.; -dms, -DMS, --DMS \| Specify a file with normalized DMS reactivity data. These data will be applied as a pseudoenergy restraint. The data are specified in a file using the SHAPE data file format.; -dsh, -DSH, --DSHAPE \| Specify a differential SHAPE data file to be used to generate restraints in addition to SHAPE restraints specified by --SHAPE. These restraints specifically use SHAPE pseudoenergy restraints where the offset is zero. Also, the function for calculating the pseudo free energy for nucleotide i is = (differential slope) \* (differential SHAPE for nucleotide i). These pseudoenergies are added to those generated with the --SHAPE option. Default is no differential SHAPE data file specified.; -dsm, -DSM, --DSHAPEslope \| Specify a slope used with differential SHAPE restraints. Default is 2.11 kcal/mol.; -dso, -DSO, --doubleOffset \| Specify a double-stranded offset file, which adds energy bonuses to particular double-stranded nucleotides. Default is to have no double-stranded offset specified.; -l, -L, --loop \| Specify the maximum number of unpaired nucleotides in an internal or bulge loop. Default is 30 unpaired nucleotides.; -m, -M, --maximum \| Specify a maximum number of structures. Note that suboptimal structures are generated until either the maximum number of structures are reached or the maximum percent difference is reached (below). Default is 20 structures.; -md, -MD, --maxdistance \| Specify a maximum pairing distance; that is, the maximum number of bases between the two nucleotides in a pair. Default is no restriction on the distance between pairs.; -mfe, -MFE, --MFE \| Specify that only the minimum free energy structure should be generated. This saves about half the computation time, but provides less information. The -p and -m options are ignored. Also, no save files can be generated using -s.; -p, -P, --percent \| Specify a maximum percent difference in folding free energy change for generating suboptimal structures. For example, 20 would indicate 20%. Default is determined by the length of the sequence.; -s, -S, --save \| Specify the name of a save file, needed for dot plots and refolding. Default is not to generate a save file.; -sh, -SH, --SHAPE \| Specify a SHAPE data file to be used to generate restraints. These restraints specifically use SHAPE pseudoenergy restraints. Default is no SHAPE data file specified.; -si, -SI, --SHAPEintercept \| Specify an intercept used with SHAPE restraints. Default is -0.6 kcal/mol.; -sm, -SM, --SHAPEslope \| Specify a slope used with SHAPE restraints. Default is 1.8 kcal/mol.; -sso, -SSO, --singleOffset \| Specify a single-stranded offset file, which adds energy bonuses to particular single-stranded nucleotides. Default is to have no single-stranded offset specified.; -t, -T, --temperature \| Specify the temperature at which calculation takes place in Kelvin. Default is 310.15 K, which is 37 degrees C.; -usi, -USI, --unpairedSHAPEintercept \| Specify an intercept used with single-stranded SHAPE constraints. Default is 0 kcal/mol.; -usm, -USM, --unpairedSHAPEslope \| Specify a slope used with single-stranded SHAPE constraints. Default is 0 kcal/mol.; -w, -W, --window \| Specify a window size. Default is determined by the length of the sequence.; -x, -X, --experimentalPairBonus \| Specify a text file with a two-dimensional matrix of bonuses (in kcal/mol) to apply to each residue pair, as might be obtained from a mutate/map measurement. The matrix must have the same number of rows and columns as the target RNA. Bonus is applied once to edge base pairs, twice to internal base pairs. Default is no experimental pair bonus file specified.; -xo \| Specify an intercept (overall offset) to use with the 2D experimental pair bonus file. Default is 0.0 (no change to input bonuses).; -xs \| Specify a number to multiply the 2D experimental pair bonus matrix. Default is 1.0 (no change to input bonuses). Notes about using DMS data: There are two ways to use DMS reactivity to improve the accuracy of secondary structure prediction. The first approach, explained in reference 3, is better when the DMS data are analyzed qualitatively. In this approach, the DMS-reactive nucleotides are specified as nucleotides accessible to chemical modification in a constraint file. The constraint file is then read using the -c option. The second approach is better when the DMS reactivities are quantified. This approach requires specification of normalized reactvities, using a SHAPE data file format. This file is then read using the -dms option. This approach is explained in reference 5. Notes for smp: Fold-smp, by default, will use all available compute cores for processing. The number of cores used can be controlled by setting the OMP\_NUM\_THREADS environment variable. References: 1. Reuter, J.S. and Mathews, D.H. "RNAstructure: software for RNA secondary structure prediction and analysis." *BMC Bioinformatics*, 11:129. (2010). 2. Deigan, K.E., Li, T.W., Mathews, D.H. and Weeks, K.M. "Accurate SHAPE-directed RNA structure determination." *Proc. Natl. Acad. Sci. U.S.A.*, 106:97-102. (2009). 3. Mathews, D.H., Disney, M.D., Childs, J.L., Schroeder, S.J., Zuker, M. and Turner, D.H. "Incorporating chemical modification constraints into a dynamic programming algorithm for prediction of RNA secondary structure." *Proc. Natl. Acad. Sci. USA*, 101:7287-7292. (2004). 4. Mathews, D.H., Sabina, J., Zuker, M. and Turner, D.H. "Expanded sequence dependence of thermodynamic parameters provides improved prediction of RNA secondary structure." *J. Mol. Biol.*, 288:911-940. (1999). 5. Cordero, P., Kladwang, W., VanLang, C.C., and Das, R. "Quantitative dimethyl sulfate mapping for automated RNA secondary structure inference." Biochemistry, 51: 7037-7039. (2012). | | |
| Visit The Mathews Lab RNAstructure Page for updates and latest information. | | |
